# Particle dynamics and deposition in true-scale pulmonary acinar models

**DOI:** 10.1038/srep14071

**Published:** 2015-09-11

**Authors:** Rami Fishler, Philipp Hofemeier, Yael Etzion, Yael Dubowski, Josué Sznitman

**Affiliations:** 1 Department of Biomedical Engineering, Technion – Israel Institute of Technology, Haifa; 2 Department of Civil and Environmental Engineering, Technion – Israel Institute of Technology, Haifa

## Abstract

Particle transport phenomena in the deep alveolated airways of the lungs (i.e. pulmonary acinus) govern deposition outcomes following inhalation of hazardous or pharmaceutical aerosols. Yet, there is still a dearth of experimental tools for resolving acinar particle dynamics and validating numerical simulations. Here, we present a true-scale experimental model of acinar structures consisting of bifurcating alveolated ducts that capture breathing-like wall motion and ensuing respiratory acinar flows. We study experimentally captured trajectories of inhaled polydispersed smoke particles (0.2 to 1 μm in diameter), demonstrating how intrinsic particle motion, i.e. gravity and diffusion, is crucial in determining dispersion and deposition of aerosols through a streamline crossing mechanism, a phenomenon paramount during flow reversal and locally within alveolar cavities. A simple conceptual framework is constructed for predicting the fate of inhaled particles near an alveolus by identifying capture and escape zones and considering how streamline crossing may shift particles between them. In addition, we examine the effect of particle size on detailed deposition patterns of monodispersed microspheres between 0.1–2 μm. Our experiments underline local modifications in the deposition patterns due to gravity for particles ≥0.5 μm compared to smaller particles, and show good agreement with corresponding numerical simulations.

Understanding the basic transport mechanisms of inhaled particles in the deep alveolated regions of the lungs, i.e. pulmonary acini, has broad ramifications in the context of health risk assessment^1^,^2^ (e.g. occupational and environmental exposure) and inhalation therapy^3^,^4^ (e.g. systemic delivery or local lung targeting). However, acinar particle dynamics are not trivial due to the coupling between intrinsic transport mechanisms^5^ (e.g. gravity, diffusion) and the complex convective airflows that exhibit recirculating alveolar flow topologies and stretch-and-fold patterns characteristic of chaotic mixing^6^. In particular, inhaled aerosol kinematics are influenced by irreversible low-Reynolds-number airflows, a consequence of anisotropic deformations of septal walls during cyclic breathing^7^ combined with unique anatomical structures of the acinus^8^.

A detailed characterization of particle transport mechanisms inside the pulmonary acinus requires resolving kinematic trajectories and detailed deposition patterns of inhaled aerosols under physiologically-realistic respiratory flow conditions. Yet, experimental studies of inhaled particle dynamics are scarce due to the intricate acinar anatomy and its small dimensions (i.e. the diameter of an alveolus is ∼200–300 μm in a human adult^9^) To date, there are still no means to measure airborne particle trajectories *in vivo*, and high-resolution aerosol deposition patterns in acini can only be obtained from direct visualization in sacrificed animals^9^ or from human autopsys^10^. In live humans, investigations of inhaled aerosols are commonly restricted to imaging deposition patterns of radio-labeled particles with coarse spatial resolutions^11^,^12^ (e.g. gamma scintigraphy), or alternatively conducting inhalation studies by measuring bolus dispersion upon exhalation at the mouth^13^. An experimental *in vitro* platform enabling direct observations of acinar particle trajectories and deposition patterns is thus highly desirable for investigating basic transport phenomena. One classic approach is brought with scaled-up models following hydrodynamic similarity matching; however, scaled-up setups are mostly limited to investigating a narrow range of realistic inhaled aerosol dynamics^5^,^14^,^15^ since Brownian diffusion mechanisms for submicron and ultrafine particles are altogether neglected.

In the absence of versatile experimental tools numerical investigations, including computational fluid dynamics (CFD), have been highly valuable for investigating the dynamics of inhaled aerosols in models of acinar geometries, spanning simple alveolated ducts^16^,^17^,^18^,^19^ to more complex bifurcating tree networks^20^,^21^,^22^, and more recently using anatomically-reconstructed alveolar geometries^23^,^24^,^25^ with high-resolution imaging modalities (e.g. micro-computed tomography). An overarching finding is that the deposition processes of both large (e.g. 2 μm and larger) and small (e.g. 0.1 μm and smaller) particles are relatively quick (i.e. with time scales on the order of an inhalation period or less), where kinematics are dominated by gravitational sedimentation for heavier particles and Brownian motion for smaller ones. However, for fine particles in the intermediate size range of ∼0.1 to 1 μm, transport is hypothesized to be largely affected by the local unsteady airflow patterns, leading to irreversible kinematics within alveolar cavities^18^,^26^,^27^,^28^. Despite such insight, the great majority of numerical works on aerosol kinematics have not been validated experimentally and have frequently discarded the role of Brownian motion.

Here, we present an *in vitro* platform for studying inhaled acinar particle dynamics and deposition patterns. Using microfabrication techniques, we have constructed a true-scale microfluidic acinar model that allows direct observation of airborne particle trajectories and mapping detailed deposition locations. Following in the footsteps of seminal microfluidic airway models^29^,^30^, our *acinus-on-chip* platform consists of an anatomically-inspired, multi-generation network of bifurcating airway ducts lined with alveolar cavities, where the walls are periodically deformed in a physiologically-realistic breathing-like fashion. We first track and reconstruct the trajectories of airborne smoke particles near and within alveolar cavities using high-speed imaging. Next, we capture the deposition patterns of a wide range of monodispersed microspheres (from 0.1 to 2 μm in diameter) following inhalation assays in the *in vitro* device. Together, our experimental observations give new insight into the coupling of aerosol transport mechanisms and local alveolar flow patterns that determine the dispersion and deposition of inhaled particles in acinar structures.

## Results and Discussion

### Device design

**Figure 1:**
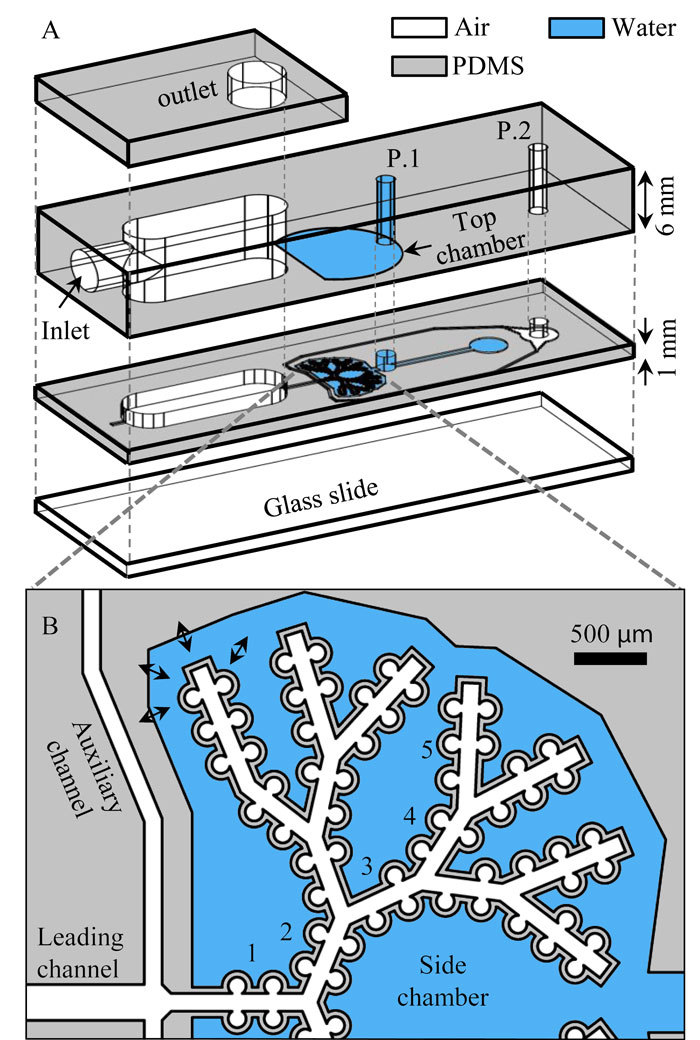
Schematic of the *acinus-on-chip* device. (**A**) Microfluidic acinar model consisting of three PDMS layers stacked on top of a PDMS-covered glass slide. (**B**) Magnified view of the acinar tree, featuring five alveolated airway generations (numbered 1 through 5). The thin airway walls, embossed in the bottom face of the bottom PDMS layer, are cyclically deformed by applying pressure to the surrounding top and side water chambers (colored in blue) via a syringe pump connected to port 1 (marked P.1). Particle tracking assays are conducted by injecting incense smoke through port 2. During deposition assays, an aerosol of polystyrene particles enters through the inlet and leaves mostly through an exhaust tube connected to the outlet. A syringe pump connected to port 2 (marked P.2) draws a slow stream of aerosols through the leading channel and auxiliary channels from which the aerosol is drawn into the alveolated airways as a result of the cyclic change of airway volume. (see Methods for further details on aerosol exposure assays).

The *acinus-on-chip* geometry is designed to mimic the pulmonary acinar environment, capturing representative alveolar structures at true scale (Fig. 1A). At the heart of the design is an anatomically-inspired model featuring five generations of rectangular ducts (120 μm in width and 100 μm in height) lined with cylindrical alveoli (120 μm in diameter; see Fig. 1B), constructed using well-established microfabrication techniques for Polydimethylsiloxane-based (PDMS) microfluidics^31^. Two master wafers were fabricated for PDMS molding using either SU-8 photolithography^31^ or deep reactive ion etching (DRIE) of a silicon on insulator wafer^30^,^32^; further details on device fabrication are presented in the supplementary information (SI). The thin PDMS airway walls are deformed in a periodic fashion with a 4 sec breathing period (*T*) by altering the pressure inside the surrounding top and side chambers (Fig. 1A) via a syringe pump (see Video S1). This results in a maximal deflection of ∼20 μm for the upper channel wall and ∼10 μm for the side walls, thus reproducing a normal to deep breathing scenario with a maximum total volume change of ∼30% of the minimum airway volume, where the tidal front reaches approximately up to the bifurcation point between the 4^th^ and the 5^th^ generations (see Fig. 1B and Videos S1 and S2). To characterize convective flow within the device, velocity profiles in acinar ducts and velocity maps of alveolar flow fields were resolved by micro-particle image velocimetry (μPIV) measurements using liquid-suspended particles and hydrodynamic similarity matching^30^ (see Methods).

The anatomically-inspired microfluidic acinar models differ in many ways from the anatomical *in vivo* environment^30^. Importantly, the alveolar to ductal volume ratio is much lower than expected anatomical values due to intrinsic microfabrication constraints^30^. In addition, airducts (120 μm in width and 100 μm in height) and alveoli (120 μm in diameter and 100 μm in height) are slightly smaller compared to anatomical data for adults^9^ such that our designs may be closer to acinar geometries of infants^33^. Nevertheless, it is stressed here that the underlying acinar airflow phenomena under breathing conditions are adequately captured in the present true-scale models. Namely, both local alveolar flow patterns and velocity flow magnitudes along the acinar ducts (see Methods) show consistent agreement with recent numerical simulations^5^,^34^. Hence, our model supports a detailed and systematic study of the basic acinar transport phenomena anticipated under respiratory acinar flow conditions^5^.

### Visualization of incense particle trajectories

We first explored how the fate of inhaled aerosols passing near an alveolus is affected by the time of arrival (e.g. inhalation or exhalation), sedimentation, diffusion and the local unsteady alveolar flow configuration.

**Figure 2:**
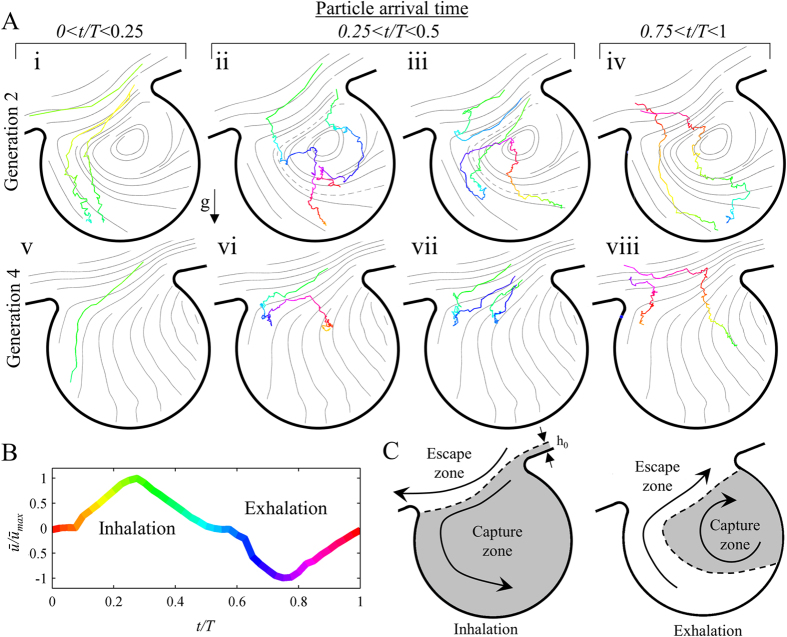
Kinematics of inhaled incense smoke particles. (**A**) Trajectories of individual particles. Color-coding indicates time in breathing cycle, i.e. red and light blue correspond to transition from exhalation to inhalation and from inhalation to exhalation, respectively. Gray lines depict airflow streamlines at maximal velocity (i.e. peak inhalation), as obtained from micro-PIV measurements (see Methods). (**B**) Normalized average velocity versus normalized time at the opening region of an alveolus in the second generation, as measured from PIV. Color-coding is identical to panel (**A**). (**C**) Schematic framework for predicting particle fate near an alveolus in the mid-acinus (i.e. second generation in our model). During inhalation (left panel), a streamline crossing the threshold distance (*h*_*0*_) defines regions of particle capture or escape. At exhalation (right panel), the recirculation region constitutes a capture zone surrounded by an escape zone. Gravity and diffusion may divert a particle from an escape zone to a capture zone (or vice versa), in particular during flow reversal and inside the alveoli where convective flows are weak.

Here, we have visualized time-resolved trajectories of airborne particles inside the *acinus-on-chip* using a dense incense smoke (∼10^9^ cm^−3^) to allow imaging of particles in a small field of view over short time scales. Following measurements of particle size distribution, the aerosol is found to be polydispersed, exhibiting an approximate log normal distribution with a count mean diameter of 0.46 μm and a geometric standard deviation of 1.25; as such, particle sizes lie in the range of ∼0.2 to 1 μm. Notably, while the range of examined particle sizes is relatively narrow, it corresponds to the size range where our current understanding of particle dynamics is most limited, as discussed further below. Microfluidic devices were held vertically with the leading channel aligned with the direction of gravity, and a plume of incense smoke was injected through port 2. Particles were then imaged using dark field microscopy (see Videos S3-S5) and their location was identified using an in-house particle tracking algorithm. Selected aerosol trajectories in the vicinity of and within alveolar cavities are shown in Fig. 2, where individual trajectories are color-coded according to the breathing cycle (Fig. 2B). Instantaneous streamlines of the alveolar flow patterns at peak velocity (*t*/*T* ∼ 0.25) are overlaid for clarity; note that the model deformation is not strictly self-similar due to intrinsic heterogeneities in acinar airway strains, and therefore alveolar flow topologies may slightly evolve and differ from those plotted during the course of the breathing, as we have previously shown^30^. Although our visualizations of individual trajectories (*n* > 40) are restricted to 2D projections only, significant out-of-plane motion occurs due to a combination of the 3D nature of the acinar flow field^24^,^30^ and intrinsic Brownian diffusion for particles in this size range. In turn, some imaged particles disappeared from the focal plane during measurements (Fig. 2).

**Figure 3:**
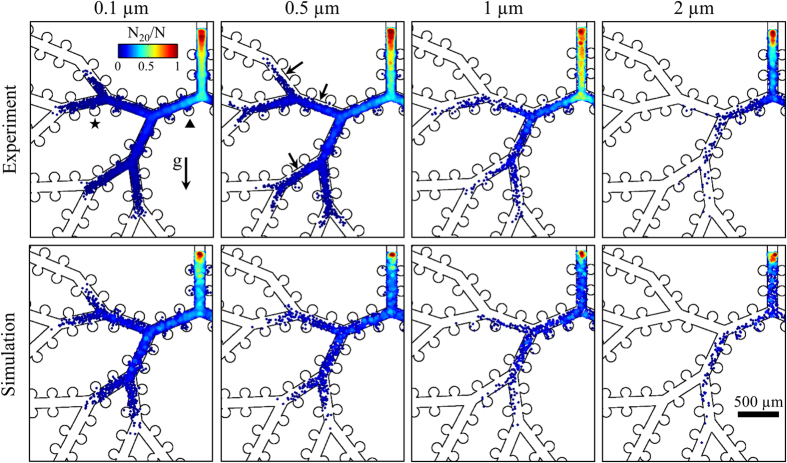
Deposition maps for aerosol inhalation experiments (first row) and corresponding numerical simulations (second row). Particle deposition locations are color-coded according to normalized particle density obtained within a circle of 20 μm radius centered on a given particle normalized by total deposition (N_20_/N). The experimental results represent cumulative data of *n* = 5, 6, 9 and 5 independent experiments, for *d* = 0.1, 0.5, 1 and 2 μm, respectively. Exposure time ranged from 1h to 3h, depending on assays. Here, alveoli imaged during particle tracking experiments (see Fig. 2) are marked by a triangle and a star (first column), and regions depleted of particles are marked with arrows (second column).

Figure 2A compares aerosol trajectories that reach the vicinity of the alveolar opening at different times during the inhalation cycle for two selected alveoli located in the second (first row) and fourth (second row) generations (marked with a triangle and a star in Fig. 3, respectively). Note that the alveolus in the second generation features a recirculating flow pattern characteristic of proximal to mid-acinar regions; these regions are acknowledged to yield relatively higher deposition relative to deeper alveoli^11^ and feature enhanced convective mixing^35^. In contrast, streamline patterns in the fourth generation are radial with no recirculation zone, where ductal velocities are significantly slower due to mass conservation across the acinar network.

We observe distinctly different deposition mechanisms as a function of particle arrival time. Early on during the inhalation phase (*t*/*T* < 0.25) smoke particles seem to behave essentially as massless tracers that may enter the alveolus by following flow pathlines leading into the alveolus (see Video S3). In contrast, particles arriving later in the inhalation phase (0.25 < *t*/*T* < 0.5) or towards the end of the exhalation phase (0.75 < *t/T* < 1), come to a near stop during flow reversal when velocities are nearly quiescent. Within this short time span (∼0.6 sec in our system, see Fig. 2B), some particles are able to cross into the alveolar cavity under the combined action of gravity and diffusion. The relative contribution of gravitational and diffusional mechanisms to this motion varies with particle size. For example, in Fig. 2A,ii the left particle is seen to follow an elongated path characteristic of larger particles where sedimentation dominates over diffusion (see Video S4). In contrast, the more meandering trajectory of the right particle suggests kinematics of a smaller particle where gravity and diffusion have similar effects.

**Figure 4:**
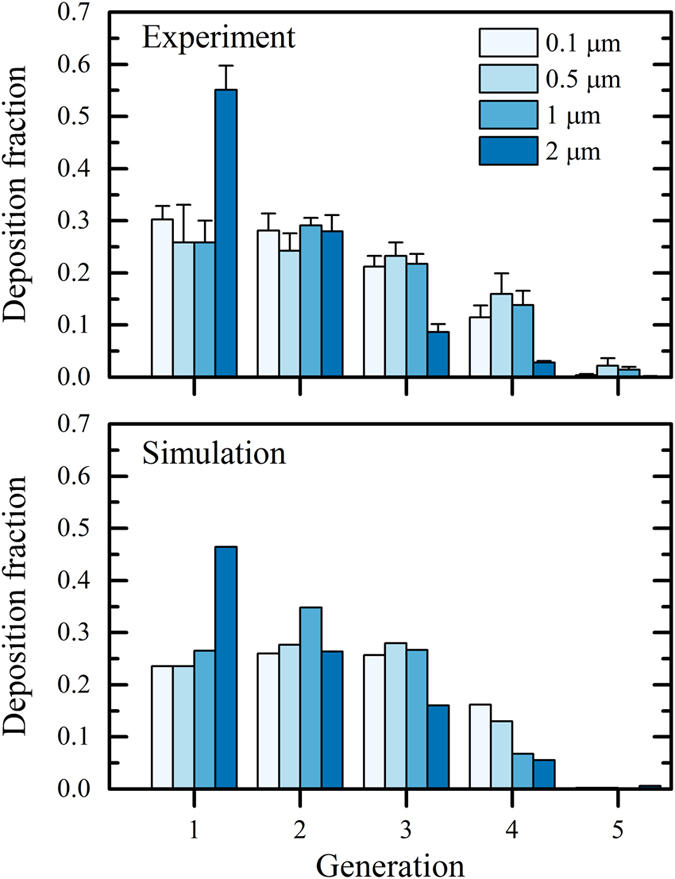
Particle deposition fraction per airway generation for experiments (top panel) and corresponding simulations (bottom panel); error bars in the top panel correspond to standard deviation (*n* = 5 to 9). A Student’s t test was used to test for statistical significance (**p < 0.005, ***p < 0.001).

While the roles of gravity for deposition of particles >1 μm and diffusion for  < 0.1 μm particles have been addressed in many numerical works^16^,^17^,^19^,^22^, the effects of these transport mechanisms on particle kinematics and deposition in the intermediate size range 0.1–1 μm still remain poorly understood. In particular, Brownian diffusion is frequently neglected in numerical simulations^20^,^22^,^27^ following high particle Peclet numbers assessed only in acinar ducts for average flow velocities, where convective transport dominates^5^. Our experiments suggest in contrast a radical change in particle kinematics inside alveoli as well as during flow reversal due to the weak convective flows. While particles in acinar ducts during peak inhalation follow closely the streamlines (Fig. 4A,i), convective oscillatory flows give rise to particle streamline crossing (Fig. 2A,ii-iv and vi-viii), a key mechanism leading to the coupling between intrinsic particle transport phenomena and acinar flow topologies.

To better understand how streamline crossing affects particle deposition, we present a simple framework for predicting the fate of particles in the vicinity of an alveolus due to the coupled effects of flow phenomena and intrinsic particle transport. Figure 2C summarizes schematically the effect of local flow patterns on particle fate in the vicinity of an alveolus in the mid-acinus (second generation of our model). Namely, during the inhalation phase (left panel), the streamline passing at a threshold distance (*h*_*0*_) from the alveolar opening separates the flow into a capture zone, drawing particles into the alveolus and an escape zone where particles are convected towards deeper acinar regions (Fig. 2A,i); the right panel of Fig. 2C depicts the corresponding capture and escape zones during exhalation. Here, the recirculation region defines a capture zone while the region outside this vortex constitutes an escape zone. The fate of airborne particles can now be anticipated by considering how gravity and diffusion may divert an inhaled particle from an escape zone to a capture zone, or vice versa. For example, the particles in Fig. 2A,ii and iv cross from an escape zone to a capture zone during flow reversal. In Fig. 2A,iii both particles are located in an escape zone at the beginning of exhalation. However, while the upper particle escapes the alveolus during the exhalation phase, the lower particle crosses into the recirculation zone due to gravity and diffusion and eventually deposits inside the alveolus. We postulate that particles passing very close to the boundary between capture and escape zones may deposit on the alveolar opening region (i.e. the corner between duct and alveolus). However, due to strong light reflections from the walls we were not able to visualize deposition in this region.

The differences in flow topologies in the deep acinus compared to the mid-acinus modify the schematic description of Fig. 2C for deeper acinar regions (e.g. generation 4 in our model). During the inhalation phase, *h*_*0*_ is larger since the ratio of ductal to alveolar flow rate is lower^18^, resulting in a wider band of streamlines that reaches into the alveolus; thus, particles may enter the alveolus when passing at a greater distance from the alveolar opening compared to generation 2 (compare Fig. 4A,i and 4A,v). Furthermore, since there is no recirculation zone, only an escape zone exists during the exhalation phase. As a result, particles that reach relatively deep into the alveolus may still escape during exhalation (Fig. 2A,vii).

It is important to note that a particle located inside an escape zone at the beginning of exhalation does not necessarily escape the alveolus during the exhalation phase (see Fig. 2A,iii, Fig. 2A,iv and Video S5). This may result from the effects of gravity and diffusion during the exhalation phase. In addition, streamline crossing during inhalation and flow reversal may bring the particle on a pathline that is too slow to carry it out of the alveolus during the exhalation.

By carefully mapping escape and capture zones and considering the detailed effects of gravity and diffusion leading to streamline crossing, it is possible to interpret and anticipate deposition outcomes inside the acinus. We further examine how our observations may help understanding the effect of gravity and diffusion on particle dispersion in the acinus. It has long been recognized that fine inhaled particle boluses are dispersed in the acinar region^14^ despite low-Reynolds-number airflows. Several factors may contribute to this dispersion. First, convective pathlines are not necessarily reversible as a result of geometrical hysteresis leading to chaotic flows^6^,^28^,^30^. Second, although the effects of gravity and diffusion are small, they may have a significant effect on particle dispersion as shown in dispersion assays under normal and micro-gravity^36^. In particular, the effects of gravity and diffusion may divert a particle from one streamline to another; if the new streamline is slower or faster on average from the original streamline, this would result in considerable dispersion of the inhaled bolus^37^. Our direct measurements of time-resolved kinematics show that mechanisms of gravitational and diffusional streamline crossing not only take place, but are critical inside alveoli and during flow reversal (e.g. Fig. 2A,iii and vii).

### Mapping deposition patterns of inhaled aerosols

We next investigated the influence of particle diameter (*d*_*p*_ = 0.1 to 2 μm) on aerosol deposition patterns inside the acinar network under cyclic breathing conditions. In each experiment, a device was positioned vertically (Fig. 3), and exposed for 1 to 3 h to a monodisperse aerosol of fluorescent polystyrene microspheres with nitrogen as the carrier gas which was fed to the inlet (Fig. 1). Individual particle locations were then determined from fluorescence microscopy and image analysis (see Methods). Cumulative results of independent experiments (*n* = 5 to 9, depending on particle size) are presented in Fig. 3 (top row), where the total number of deposited particles ranges from ∼1,200 for 2 μm particles to ∼29,000 for 0.1 μm particles. Here, particles are color-coded according to the normalized particle density (ranging from 0 to 1), representing the number of particles located within a radius of 20 μm from a given particle (N_20_) normalized by the total number of deposited particles (N). Note that due to limitations of the current imaging technique (see SI), we plot only the particles which are deposited on the face of the airways adjacent to the glass slide (Fig. 1A). For comparison, the bottom row of Fig. 3 shows CFD simulations of particle deposition in the acinar network geometry under idealized self-similar breathing conditions (see Methods for further details).

A first and striking observation is the steep decline seen in the local concentration of deposited particles towards distal generations, i.e. the last generation is almost void of particles independent of the particle size investigated. This result is in line with *in vivo* experiments^11^ and numerical simulations^20^ where inhaled particles deposit preferentially in terminal bronchioles and proximal acinar generations. Our results underline the basic challenge in delivering inhaled aerosols (e.g. systemic delivery) to the deep acinar regions since approximately half of the alveolar surface resides within the last acinar generation^38^. The results of Fig. 3 also show a pronounced effect of particle size on deposition patterns. To help interpret this effect, Table1 summarizes the characteristic settling lengths (*l*_*s*_) and 2D diffusional lengths (*l*_*D*_) traveled in one second for polystyrene microspheres both in nitrogen (i.e. the carrier fluid in our deposition experiments) and in air at 25 °C. Here, we assume a 2D Brownian displacement for our analysis since our microscopy visualizations are limited to 2D deposition patterns on a planar wall, or alternatively 2D projections of particle trajectories in the field of view. The values presented in Table 1 show that the lower viscosity of pure Nitrogen compared to air results in slightly shorter settling and diffusional lengths; these differences are within 3.4% for settling lengths and 1.7% for 2D diffusional lengths. Note that inertial effects are negligible since the Stokes number (*Stk*), relating inertial to viscous effects, is always smaller than 0.002. Further details on the calculation of *l*_*s*_*, l*_*D*_, and *Stk* are provided in the SI.

We begin by noting that 0.1 μm particles are almost homogenously distributed along the width of the airways as anticipated from their slow sedimentation process (*l*_*s*_ = 0.86  μm in N_2_) and relatively high diffusive nature (*l*_*D*_  = 51.3 μm in N_2_). For larger 0.5 μm particles, the region near the upper side of the third and fourth generations (Fig. 3, marked with arrows) is depleted of deposited particles. Such deposition patterns result from the non-negligible settling length (*l*_*s*_  = 10.7 μm in N_2_), Similar in magnitude to the competing diffusional length (*l*_*D*_ = 16.2 μm in N_2_). 1 *μ*m particles show an even greater gravitational shift due to an increased sedimentation length (*l*_*s*_  = 37.5 μm in N_2_) along the vertical direction. As a result, such particles are not seen to reach into the top branch of the fourth generation. For larger 2 μm particles, where *l*_*s*_ = 139 μm in N_2_, the effects of gravity dominate even more and few particles reach altogether the top regions of the airway tree.

The effects of both diffusion and gravity on local deposition patterns also underline the ability of particles to cross into alveoli and deposit within. For example, 0.1 μm particles are seen to deposit inside alveoli where gravity is directed away from the alveolus suggesting an important role for diffusion. In contrast, 0.5-2 μm particles are almost always deposited inside alveoli where gravity points into the alveolus highlighting the well-known importance of sedimentation for alveolar deposition^17^. Such observations are in excellent agreement with our results for particle trajectories (Fig. 2), where gravity was seen to play a crucial role in determining alveolar deposition.

Generally, our numerical results show very good agreement with experiments, although in the simulations a steeper decline in particle concentration is noted in the vicinity of the entrance region (Fig. 3). A possible explanation for such discrepancy is that while a constant volume concentration is injected at the inlet in our simulations, the experimental particle concentration near the wall is expected to be lower than in the center of the entrance duct due to deposition in the leading channel. Despite differences in boundary conditions, the good agreement between experiment and simulations represents a first of a kind experimental validation of numerically obtained acinar deposition patterns.

To further quantify deposition, we extract the relative amount of deposited particles according to airway generation for experiments (Fig. 4, top panel) and simulations (Fig. 4 bottom panel). Overall, comparable depositional trends are observed for experimental and numerical results. Namely, particles with a diameter of 0.1, 0.5 and 1 μm show similar generational deposition fractions, where 20–30% of the particles deposit in the first generation with a similar fraction in the second and third generation. This is followed by a decline in deposition fractions towards the fifth generation. However, 2 μm particles show a bias towards proximal generations with significantly increased deposition fractions in the first generation, as well as decreased levels of deposition in the third and fourth generations. Our experimental and numerical results are in line with previous numerical works^17^,^22^,^25^, showing that for particles equal or larger than 1 μm gravity plays a major role. Furthermore, our observations corroborate the underlying effects of sedimentation observed in inhalation studies *in vivo*, where for example micro-gravity experiments^5^ show a pronounced decrease in deposition efficiencies of 1 μm and 2 μm particles compared to normal gravity conditions. Generally, the small discrepancies observed between experiments and simulations (Fig. 4) highlight a few effects that could not be resolved using the experimental scheme. For example, it appears that for 1 μm particles, maximal deposition is achieved in the second airway generation. In addition, deposition in the fourth generation (Fig. 4, bottom panel) appears as inversely correlated to particle size where deposition fractions are highest for the smallest particles (0.1 μm) and lowest for the largest particles (2 μm). This latter observation underlines the importance of gravitational deposition in proximal generations and diffusional deposition in distal regions.

An important limitation of the current experimental setup is that a classic deposition metric such as the deposition efficiency cannot be directly assessed since particles entering and exiting the model cannot be accurately counted. Moreover, even in the event where such assessment could be achieved, the small alveolar to ductal volume ratio limits the straightforward translation of our system for deposition efficiency analysis with respect to *in vivo* conditions. Under such restrictions, we thus confined our investigation to the study of basic transport phenomena (Fig. 2) and deposition processes (Figs 3 and 4).

## Conclusion

We have developed a true-scale, anatomically-inspired model of the lung acinus featuring a bifurcating tree of alveolated airways with moving walls. Despite the simplicity and limitations of such model compared to the true *in vivo* acinar environment (see also SI), our experimental platform offers analysis of detailed deposition patterns under a controlled aerosol exposure environment, and importantly a modality enabling time-resolved imaging of inhaled airborne particles within representative acinar structures.

Our unique observations underline the complexity of acinar particle dynamics which result from the coupled effects of gravity, diffusion and local alveolar airflow patterns. In particular, examination of individual inhaled particle trajectories suggest a comprehensive framework for determining the fate of particles in the vicinity of alveoli of proximal generations: (i) during inhalation, a threshold distance from the alveolus opening defines a capture zone for alveolar deposition, whereas (ii) during exhalation, the alveolar recirculation region constitutes a capture zone surrounded by an escape zone; in addition, (iii) gravitational and diffusional effects lead to a streamline crossing mechanism that allows particles to pass between capture and escape zones, specifically during flow reversal and within alveoli and has a major contribution towards particle dispersion outcomes in the acinus. Furthermore, our detailed deposition maps suggest significantly altered deposition patterns of 0.5–2 μm particles compared to 0.1 μm particles, as a result of diffusional and gravitational effects.

## Methods

### Flow characterization using micro particle image velocimetry (PIV)

To visualize the flow patterns expected inside our device, the airways were filled with a 64/36 (v/v) glycerol/water mixture seeded with red fluorescent polystyrene particles 0.86 μm in diameter (Fluoromax, Thermo Scientific). Since this solution has approximately the same kinematic viscosity as air at ∼24 °C (*ν*_*air*_ = 1.55 × 10^−5^ m^2^/s, ν_*glycerol/water mixture*_ = 1.51 × 10^−5^ m^2^/s), hydrodynamic similarity is closely matched (i.e. Reynolds number). Cyclic breathing flows were visualized using a commercial micro-particle image velocimetry (μPIV) system (Flow Master MITAS, LaVision GmbH) and flow maps were calculated using a sum of correlation algorithm on phase locked double frames. For further details on the micro-PIV method, the reader is invited to consult details pertaining to first prototype of the acinus-on-chip device^30^.

In the present study, unlike the first *acinus-on-chip* prototype, the channel width was reduced from 345 μm to 120 μm to achieve a more square-like rather than rectangular cross-section (see Fig. 1B). In addition, the long extensions in generation 5 were eliminated. We therefore conducted a thorough flow characterization in the new designs. Here, the Reynolds number, average flow velocities, and ductal to alveolar flow ratios inside the device are very similar to our previous results, and agree well with expected values in the mid- to distal acinar generations. Flow profiles across the width of the channels (Fig. S1) are observed to be more parabolic compared to the plug-like character in the former design due to the smaller aspect ratio of the ducts. Velocity magnitude as function of time was measured near the opening of an alveolus in generation 2 as shown in Fig 2B. Figure S2 shows alveolar flow patterns at peak inhalation inside alveoli spanning generations 1 to 5.

### Visualization of incense particle trajectories

To visualize airborne incense particles inside the *acinus-on-chip*, the device was positioned with the leading channel aligned with the direction of gravity (Fig. 4), during which the device walls were continuously actuated to mimic breathing motion. An incense stick (Satya, Shrinivas Sugandhalaya, India) was lit and laid flat on an aluminium foil, and the flame was allowed to extinguish. Immediately afterwards, the dense smoke plume was drawn into a 5 ml plastic syringe and injected into the device through port 2 (Fig. 1A) until smoke was visibly exiting the device through the inlet and outlet. This resulted in a dense aerosol residing inside the leading channel without flow, allowing a dense stream of particles to enter the alveolated airways upon expansion of the device walls within one or two breathing cycles. This exposure method was preferable for particle visualization compared to the exposure method used for particle deposition studies, since particle density inside the alveolated airways was maximized during the short visualization time spans for particle tracking. Airborne incense particles were imaged at 50 frames per second (fps) using a 20X objective with a numerical aperture of 0.4 (LD-plan Neofluar, Zeiss) and a high-speed CMOS camera (HighSpeedStar, LaVision). The device was illuminated at 90 degrees to the imaging angle using a halogen light source (Ace 1, Schott) resulting in the capture of dark field image sequences (592 × 558 pixels) where particles appear bright on a dark background. Image post-processing steps included background subtraction using the average local intensity of the image sequence followed by smoothing using a 9 × 9 pixel Gaussian filter. Particle location was then determined as the intensity weighted centroid of pixels that have an intensity above a predefined threshold value.

### Size distribution measurement for incense particles

The size distribution of incense smoke particles was measured using an aerosol spectrometer (Droplet Measurement Technologies, model PCASP-X2), as shown in Fig. S3. Smoke particles were collected using a 5 ml syringe from either (i) a smoldering incense stick or (ii) immediately after the flame was extinguished. The size distribution for the two sampling methods was dramatically different. While particles emitted from a smoldering incense stick were generally smaller than 0.6 μm where most of the particles are smaller than 0.3 μm in accordance with previously reported data^39^,^40^; particles collected immediately after extinguishing were significantly larger, exhibiting an approximate log normal distribution (see best fitting Gaussian curve in Fig S3) with a count mean diameter of 0.46 μm and a geometric standard deviation of 1.25. This difference is most likely caused by coagulation of particles due to the larger concentration of particles upon extinguishing of the flame. We conclude that the incense particles visualized inside the device are likely to range from 0.2 to 1 μm in diameter although further growth due to higher pressure and humidity inside the device cannot be ruled out. Note that here background particles present in the room were not reduced from the size measurement since the interest in our measurement is to determine particle size distribution inside the model (which includes also background particles) and not the properties of incense smoke particles alone.

### Mapping deposition patterns of polystyrene particles

A monodispersed aerosol was produced by aerosolization of water suspended polystyrene red fluorescent microspheres (Fluoromax red fluorescent microspheres 1% solid, Thermo Scientific) using an aerosol generator (model 3076, TSI) with nitrogen as the gas source and subsequently drying the water droplets using two consecutive diffusion driers (model 3062, TSI). The aerosol flow rate was reduced by splitting the aerosol stream into two tubes, one leading outside of the lab and the other to the model. Before entering the model, the aerosol was passed through a charge neutralizer (TSI 3077) to minimize electrostatic effects. The overall flow rate through the device was 360 cc/min as measured using a flow meter at the aerosol outlet of the device. Since the volume of aerosol entering the alveolated airways of the model in each breath cycle is very low (∼0.05 mm^3^), relatively high aerosol concentrations had to be used (∼10^6^ particles/cm^3^) in order to obtain a significant amount of deposited particles in reasonable time (up to a few hours). Nevertheless, it was necessary to avoid the formation of aerosol aggregates due to the high concentrations of water suspended particles^41^. In accordance with these considerations the microspheres were dispersed in water by sonication for 20 min. The final weight percentages of the particle suspensions were 0.05% for 0.1 μm particles, 0.1% for 0.5 and 1 μm particles and 0.2% for 2 μm particles. These concentrations result in an aerosol with less than 1% aggregates assuming that the water droplets exit the atomizer with a number mean diameter of 0.35 μm and a geometrical standard deviation of 2 as specified by the manufacturer. Here, we follow the analysis of Rabbe^41^ which assumes a log-normal distribution of droplet sizes and monodispersed spherical particles.

In each experiment, a microfluidic device was positioned in a vertical orientation, where the leading channel was aligned with the direction of gravity (see Fig. 4). The aerosol was fed to the aerosol inlet through an antistatic tube and the device walls were actuated for 1 h while a steady flow of aerosol was drawn at 72 μl/min through the leading and auxiliary channels using a syringe pump connected to port 2. This configuration helped increase the amount of particles reaching the airway model and minimize loss of particles due to impaction, by bringing a slow steady stream of aerosol in close proximity to the first airway generation. During the experiment the device actuation was stopped every 10 minutes, pressure inside the device was released using a 3 way stop cock positioned between the water syringe and port 1, and then actuation was restarted. This maneuver prevented the accumulation of excess pressure in the water chamber during the long actuation period.

For 2 μm particles, it was difficult to obtain a sufficient amount of deposited particles using the above protocol due to the relatively low aerosol concentration and clogging at the channel entrance. Therefore, we used a modified method for exposing the model to 2 μm particles. Aerosols were not passed through a charge neutralizer and the aerosol stream was split immediately before entering the device. In addition, the entrance to the leading channel was cleaned from particles every 10 min using Patafix (UHU), and the overall exposure time was extended to 3 h.

After aerosol exposure, the device was examined using an inverted fluorescent microscope at 10X to 60X magnification depending on the size of the particles. A single image of the complete airway model was created by tiling multiple images and the location of each deposited particle was found by locating local intensity maxima using ImageJ software.

### Computational methods

The numerical simulations were performed using a customized solver in OpenFOAM (version 2.1.1), as recently published^42^. Briefly, the Navier-Stokes equations (i.e. mass and momentum conservation for the air) were solved using a finite-volume approach in an arbitrary Lagrangian Eulerian (ALE) framework^43^,^44^. In order to ensure high spatial and temporal accuracy second-order discretization schemes, e.g. Crank Nicholson and central differencing scheme, were implemented^42^. Particle dynamics (i.e. conservation of linear momentum) were modelled in a Lagrangian framework undergoing convection (i.e. viscous drag), sedimentation and diffusion forces. Due to low local particle concentrations, a one-way fluid-particle coupling scheme was chosen (i.e. particles do not influence the resulting flow field), whereby inter-particle effects were neglected.

The airway geometry of the exact microfluidic device (Fig. 1) was discretized with approximately 580,000 hexahedral cells providing a sufficient discretization, as prior mesh-sensitivity tests have shown. Here, a local mesh refinement was performed in the vicinity of the surfaces and in particular near the alveolar openings. The kinematic breathing motion was modelled as a cyclic, self-similar expansion of the domain^6^,^18^,^28^,^45^,^46^ where wall motion is achieved by scaling all model dimensions from a reference point, i.e. origin, by a periodic time-dependent factor. Note that such self-similar expansion is not identical to the geometrical changes in the experimental model (see Limitations section in the Supplementary Information). Temporally, the expansion of the computational domain follows the average inlet velocities of the experimental measurements as shown in Fig. 2B, where the maximal volume in the simulations are matched to the nominal dimensions of the maximal experimental volume. Accordingly, the domain volume expands by ∼33% of the minimal volume during the breathing cycle. During the first inhalation phase, ∼30,000 particles (*d*_*p*_ = 0.1, 0.5, 1, 2 μm) were continuously injected at the inlet with a constant volume concentration and tracked over two breathing cycles to ensure complete deposition. For further details on the numerical scheme and particle transport modelling see Hofemeier and Sznitman^42^.

## Additional Information

**How to cite this article**: Fishler, R. *et al.* Particle dynamics and deposition in true-scale pulmonary acinar models. *Sci. Rep.* **5**, 14071; doi: 10.1038/srep14071 (2015).

## Supplementary Material

Supplementary Information

Supplementary Video S1

Supplementary Video S2

Supplementary Video S3

Supplementary Video S4

Supplementary Video S5

## Figures and Tables

**Table 1:** Comparing the role of sedimentation and diffusion.

	**Settling length in 1 sec (μm),** ***l***_***s***_		**Diffusion length in 1 sec (μm),** ***l***_***d***_	
**particle diameter (μm)**	**Nitrogen**	**Air**	**Nitrogen**	**Air**
0.1	0.86	0.84	51.3	50.5
0.5	10.7	10.4	16.2	15.9
1	37.5	36.3	10.7	10.5
2	139	135	7.29	7.17
