# Supplementary material for: Particle dynamics and deposition in true-scale pulmonary acinar models
Source: Sci Rep. 2015 Sep 11;5:14071. doi: 10.1038/srep14071 (PMC4566083; doi:10.1038/srep14071)
Supplement: Supplementary Information [file srep14071-s1.pdf]

Supplementary Information

Particle dynamics and deposition in true-scale pulmonary acinar models

Rami Fishler*, Philipp Hofemeier*, Yael Etzion†, Yael Dubowski†, and Josué Sznitman*[1]

*Department of Biomedical Engineering, Technion – Israel Institute of Technology, Haifa.

†Department of Civil and Environmental Engineering, Technion – Israel Institute of Technology, Haifa.

Fabrication of master wafers

Two 4" master wafers were fabricated. The first wafer was made using standard SU-8 photolithography and served as a template for creating the top chambers in the middle PDMS layer (see Fig. 1A, main text). A clean 4" silicon wafer (SVM) was used without further cleaning or drying. SU-8 2075 photoresist (Microchem) was spin coated on the wafer at 500 rpm for 10 s with a 100 rpm/s ramp followed by 2000 rpm for 30 s with a 300 rpm/s ramp to a height of ~100 µm. The wafer was then soft-baked at 65°C for 5 min and at 95°C for 25 min on two separate hot plates. UV exposure was performed using an MA6 mask aligner (SUSS) with an exposure energy of 230 mj/cm^2. After a post exposure bake at 95°C for 10 min on a hot plate, the wafer was developed in 1-methoxy-2-propanol acetate (PGMEA) for 5 min, washed with isopropanol and spin dried.

The second wafer served as a template for the airways and side chambers in the bottom PDMS layer (see Fig. 1A, main text). Obtaining reproducible PDMS moulds using a master wafer formed with SU-8 was rendered difficult due to the relatively high aspect ratio of the thin walls separating the airways from the side chamber (approximately 45 µm), which lead to adherence of the PDMS to SU-8 inside the grooves. We therefore opted for an alternative method for master wafer production using deep reactive ion etching (DRIE) (Plasma-Therm, Versaline) of a silicon on insulator (SOI) wafer[1]. Here, we used a SOI wafer (SVM) with a 500 µm handle layer, an 0.5 µm buried oxide (BOX) layer, a 100 ±1 µm device layer and an 0.4 µm coating silicon oxide layer. In a first step, photolithography was used to create a masking layer on top of the SOI wafer: the wafer was dried on a hot plate at 240°C for 10 min, cooled down by spinning at 4000 rpm for 60 sec and

[1] Corresponding author: sznitman@bm.technion.ac.il

then spin coated with an AZ 4562 positive resist (AZ-Electronic Materials) at 4000 rpm for 60 sec to a height of ~6 µm. Soft bake was performed at 90°C for 10 min in an oven and 1 min at 120°C on a hot plate. After cooling down to room temperature, the wafer was exposed to UV light through a transparency mask (CAD/Art Services) in an MA-6 mask aligner (SUSS) using two 4 sec exposures at 14 mW/cm^2 separated by a 20 sec wait time. Finally, the wafer was developed in TMAH 2.5% for 3.5 min, washed in water and spin dried.

Top hard mask opening of the 0.4 µm silicon oxide layer was performed by anisotropic Reactive Ion Etching (RIE) with CF_4/O_2 RF plasma. The device layer was then etched using DRIE with 175 Bosch cycles of varying duration times. This morphing process helped achieving nearly perfect vertical walls. At the end of the process, the buried oxide layer was reached, where etching is slowed by a factor of ~70 compared to the device layer. Therefore, flat-bottomed trenches were obtained, facilitating the moulding of flat-bottomed PDMS walls that can be tightly sealed onto smooth surfaces. The last step in the process was a passivation step with a C_4F_8/Ar gas mixture leaving a fluorocarbon anti-sticking layer on the surface. This layer prevents adherence of PDMS to the master wafer during device production enabling repeated use of the master wafer (i.e. at least 30 times). The detailed DRIE process parameters are summarized in Table S1.

Table S1. DRIE parameters

**Parameters**	**Passivation**	**Etch**
Process time	5→4 sec	8→10 sec
Pressure	22 mTorr	22 mTorr
C4F8	60 sccm	
SF6		100 sccm
Ar	40 sccm	40 sccm
Bias RF power	1 W	17 W
ICP RF power	1250 W	1250 W
Electrode Temperature	10°C	10°C
ICP RF frequency	2MHz	
Bias RF frequency	13.56MHz	

Casting and multilayer construction of the device

The middle PDMS layer was produced by pouring a 1:10 curing agent:PDMS mixture on the SU-8 patterned master wafer to a height of ~6 mm. Next, the PDMS was degased by placing it in a desiccator under vacuum until all bubbles were removed, and then baked at 65°C on a levelled shelf for at least 2 h. Similarly, the 1 mm high middle layer was produced using the DRIE patterned wafer, and the top PDMS layer (~3 mm in height) was prepared using an un-patterned wafer. After baking, the middle PDMS layer was cut out from the master wafer using a scalpel. Then, port 1 was punched through the top chamber (see Fig. 1 A, main text) using a 1 mm biopsy punch, and the aerosol inlet and outlet were punched using a 3 mm biopsy punch. A very thin layer of a degased 1:10 curing-agent:PDMS mixture was carefully applied to the top surface of the bottom PDMS layer while this layer was still covering the DRIE patterned wafer. This mixture was manually applied while wearing a clean glove and allowed to level for ~3 min before the middle PDMS layer was deposited on top of it. Note that if the wet PDMS layer is not sufficiently thin, PDMS pillars may form connecting the bottom PDMS layer to the top face of the top chamber. These pillars, if present, cause a non-uniform deflection of the PDMS when the device is actuated and therefore should be avoided.

The two lower PDMS layers were then baked at 65°C for at least 1 h. Note that the hole that was punched through the top chamber is necessary in order to allow the expanding hot air to freely escape from the top chamber. In the next step, the two lower PDMS layers (now connected by the cured thin PDMS layer between them) were cut out from the master wafer. Port 1 was then deepened by inserting a biopsy punch through the existing hole and punching further through the bottom PDMS layer. In this manner, a single port feeds both the top chamber located in the middle PDMS layer and the side chamber located in the lower PDMS layer. Port 2 was then punched through both PDMS layers. A thin layer of a 1:5 curing-agent:PDMS mixture was spin coated on a glass slide and baked for ~15 min at 65°C. The two lower PDMS layers were then deposited on the PDMS covered glass. Next, the top PDMS layer was cut out of the un-patterned wafer and punched with a 3 mm biopsy punch to create an aerosol outlet. This layer was glued to the middle PDMS layer using a layer of 1:10 curing-agent:PDMS mixture. The complete device was then baked overnight at 65°C.

Device filling and actuation

To fill the top and side chambers with water, a drop of water was placed on top of port 1 and placed in a desiccator under vacuum for 5 min. Next, the water was positively pressurized using a water filled 1 ml syringe (Norm-Ject; Henke Sass Wolf, Tuttlingen, Germany) until all residual air inside the chambers diffused into the surrounding PDMS. The syringe was then connected to a

syringe pump (PHD Ultra, Harvard apparatus) pre-programmed to mimic a tidal breathing cycle (T=4 sec) constructed of linear ramps, i.e., from zero to Q_{max} in 1 sec, from Q_{max} to $-Q_{max}$ in 2 sec and from $-Q_{max}$ back to zero in 1 sec. Note that only positive pressure is always applied to the water chambers to avoid bubble formation. Here, Q_{max} is the maximal flow rate which was adjusted for each device individually to match a vertical deflection of 20-23 µm of the upper wall of the airways, measured by focusing on the upper wall in an inverted phase-contrast microscope. The value of Q_{max}, therefore, varied between 960 µl/min and 1300 µl/min according to the specific device resulting in a lateral wall deflection of the thin side walls (measuring 45 µm in width) by ~10 µm. This procedure minimizes the variability of volume change between different devices resulting from slight changes in PDMS properties and device preparation conditions. To evaluate the resulting volume change the alveolated airways were filled with water and the volume of water that was displaced out of the airways upon wall deformation was calculated from image analysis. The measured volume change was ~30% of the minimal airway volume, corresponding physiologically to a normal to deep breathing scenario. The tidal front was visualized by filling the alveolated ducts with water and then replacing the water in the leading channel with a water suspension of 0.86 µm polystyrene microspheres. These particles were then imaged using fluorescent microscopy during inflation of the alveolated ducts, and the tidal front was observed to reach approximately the bifurcation point between the 4th and 5th generations.

Calculation of particle transport properties

In Table 1 (main text), the settling distance in 1 sec was computed using the following equation for the terminal settling velocity, $u_t=C_c d^2 \rho g/(18\mu)$. Here, d is the particle diameter, ρ is the particle density, g is the acceleration of gravity, μ is the dynamic viscosity of Nitrogen at 25°C, and C_c is the Cunningham slip correction factor approximated as $C_c=(1+2.52\times\lambda/d)$, where λ is the mean free path for Nitrogen or air at 25°C and ambient pressure. The characteristic diffusion length in 1 sec was calculated based on the root-mean-squared (RMS) displacement in 2D expected for an ensemble of particles, $x_d=(4D_d t)^{1/2}$, where t is time, and D_d is the particle diffusion coefficient given by the Stokes-Einstein relation for a spherical particle, i.e. $D_d=k_B T C_c/(3\pi\mu d)$. Here, k_B is Boltzmann's constant and T is the absolute temperature (in Kelvin). The Stokes number, $St=\rho d^2 \bar{u}_x C_c/(18\mu w_d)$, gives an estimate of the importance of particle inertia, where $\bar{u}_x$ is the average velocity across the duct (~0.01 m/s in generation 1) and w_d is the duct width. Note that for air the dynamic viscosity is 3.4% higher than for Nitrogen whereas the mean free path is almost identical. Therefore, the Reynolds number and the corresponding non-dimensional particle numbers change by less than 5%.

This suggests that similarity matching for flow visualization and the comparison of particle trajectories to the CFD simulations (which use μ for air) remain valid.

Limitations

In addition to the main restrictions of our acinar model discussed in the article (see subsection "Device design" in "Results and Discussion"), we consider here a few more important limitations that should not be overlooked and suggest future paths for improving the device.

While it is known that a large amount of particles deposit on alveolar septa[2], we could not observe the trajectories of such particles in the current dark field microscopy setup due to strong reflections of light from the thin PDMS side walls. In addition, since incense particles are intrinsically polydispersed (see distribution profile in Fig. S3), we could not accurately estimate the size of the tracked particles and were thus limited to a gross size estimate using the measured particle size distribution. In principal, tracking fluorescent monodispersed particles can solve both of the above limitations. However, using our aerosol generator we were not able to produce an aerosol of fluorescent particles at sufficiently high concentrations for direct observation of airborne particles.

In our deposition studies, only particle deposition along the airway wall adjacent to the bottom glass slide was considered. Although these particles are the most accessible for imaging using an inverted fluorescent microscope, a large amount of particles deposit also on the side walls adjacent to the side water chamber (Fig. 1B, main text). In preliminary experiments we located these particles by filling the airways with liquid PDMS after the deposition assay (for refractive index matching), and imaging them using confocal microscopy. This approach served only for relatively small areas of the model and was hard to implement across the entire airway tree network due to the slow scanning speeds of the confocal microscope. Nevertheless, our preliminary results showed a much lower deposition rate on side walls compared to simulations. This discrepancy can be a result of the difference in wall deformation schemes as well as the effect of residual electrostatic charge in the PDMS. The effects of electrostatic charges in our system would require further investigation due to the fact that the neutralizer produces a distribution of particle charges with zero average charge rather than zero charged particles (e.g. for 1 µm particles, only 10% of the particles have zero charge). In addition, we could not use the neutralizer for 2 µm particles. Nevertheless, the generally good agreement between experiments and simulations (Figs. 3 and 4 of main text) for gravity effects on particle deposition suggests that the effects of excess charge in the PDMS does not influence sensibly the underlying aspects of particle transport inside our models. Producing devices using a

conductive polymer[3] may help reduce undesirable electrostatic effects and better mimic the relatively conductive tissue of the lung where no excess charge is anticipated.

References

1. Pihl, J. *et al.* Microfluidic Gradient-Generating Device for Pharmacological Profiling. *Anal. Chem.* **77,** 3897–3903 (2005).
2. Zeltner, T. B., Sweeney, T. D., Skornik, W. A., Feldman, H. A. & Brain, J. D. Retention and clearance of 0.9-micron particles inhaled by hamsters during rest or exercise. *Journal of Applied Physiology* **70,** 1137–1145 (1991).
3. Khosla, A. & Gray, B. L. Preparation, Micro-Patterning and Electrical Characterization of Functionalized Carbon-Nanotube Polydimethylsiloxane Nanocomposite Polymer. *Macromol. Symp.* **297,** 210–218 (2010).

Supplemental Figures

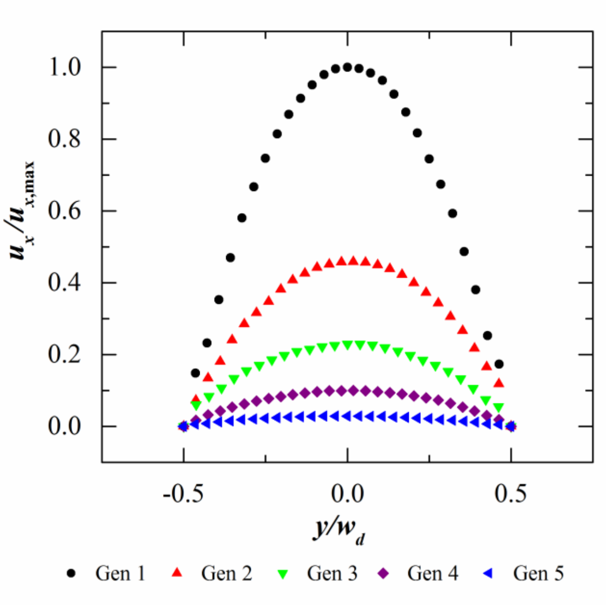

Fig. S1. One-dimensional flow profiles across the width of the channels in generations 1 to 5. $\boldsymbol{u_x}$ is the streamwise velocity at the midplane of the channel at the instant of maximal velocity, while $\boldsymbol{u_{x,max}}$= 0.016 m/s is the maximal value of $\boldsymbol{u_x}$ in generation 1. $\boldsymbol{y}$ is the spanwise coordinate and $\boldsymbol{w_d}$ is the channel width.

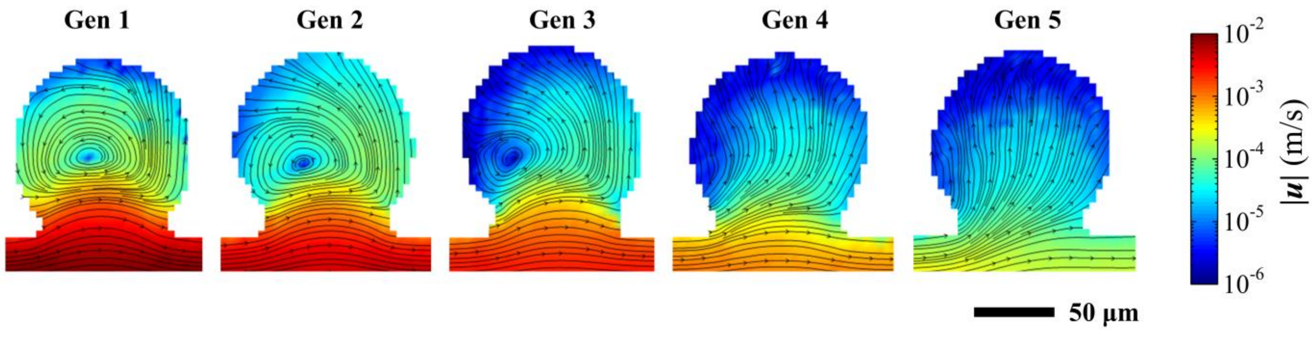

Fig. S2. Alveolar flow magnitude and corresponding streamlines obtained from micro-PIV in generations 1 to 5 at the instant of peak inhalation (t/T=0.25). Here, $\boldsymbol{u}$ corresponds to the 2D projection of the velocity field extracted at the midplane of the alveolus.

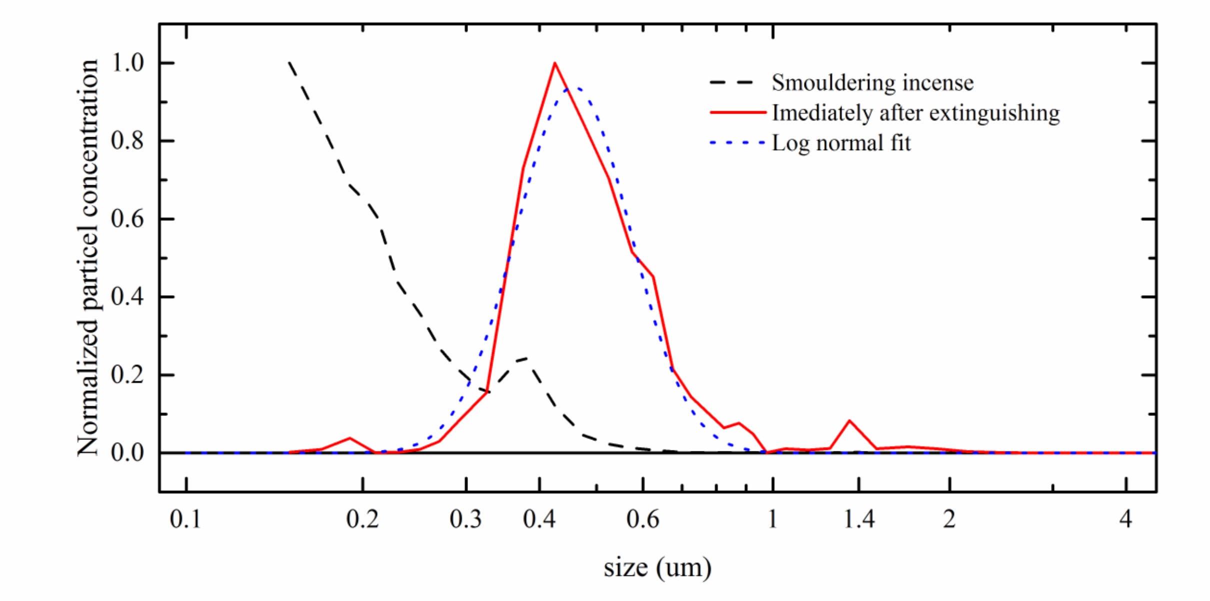

Fig. S3. Size distribution for incense smoke sampled from either a smouldering incense stick or immediately after the flame was extinguished. A log-normal best curve fit is shown for particles sampled immediately after extinguishing.
